# Supplementary material for: TRPM7 Modulates Human Pancreatic Stellate Cell Activation
Source: Cells. 2022 Jul 21;11(14):2255. doi: 10.3390/cells11142255 (PMC9316618; doi:10.3390/cells11142255)
Supplement: Supplementary file 1 [file cells-11-02255-s001.zip › cells-1756300-supplementary.pdf]

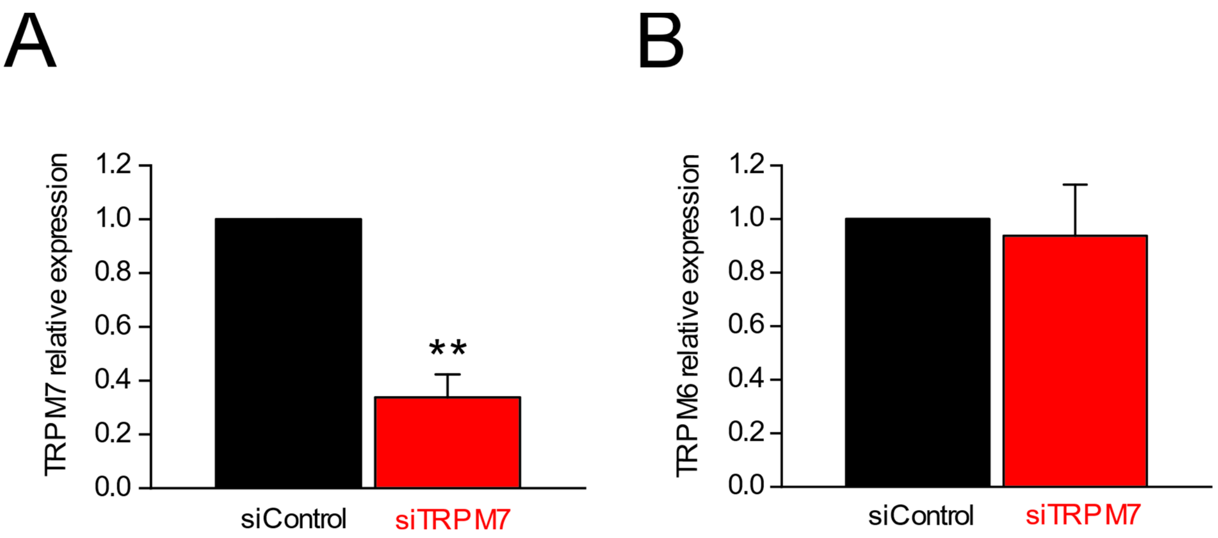

Figure S1: Effect of TRPM7 targeting siRNA on TRPM6 and TRPM7 mRNA expressions. (A) TRPM7 silencing decreased TRPM7 expression at the mRNA level (n=6). ** indicates p<0.01 (Mann-Whitney Rank Sum Test). (B) TRPM7 silencing had no effect on TRPM6 mRNA expression (n=4).

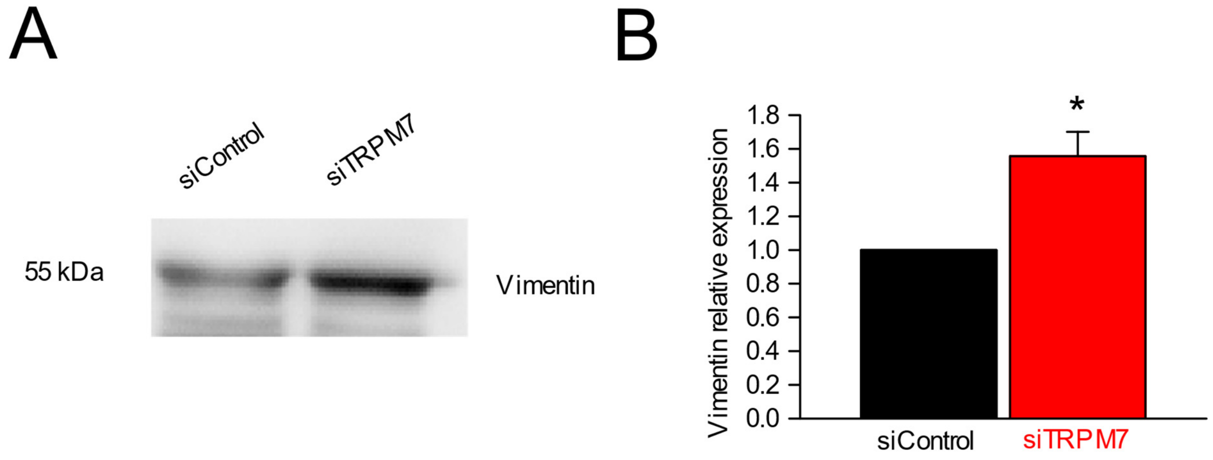

Figure S2: Effect of TRPM7 targeting siRNA on vimentin expression. (A) immunoblotting showing the effect of TRPM7 silencing on vimentin expression. (B) TRPM7 silencing increased vimentin expression (n=4). * indicates p<0.01 (Mann-Whitney Rank Sum Test).

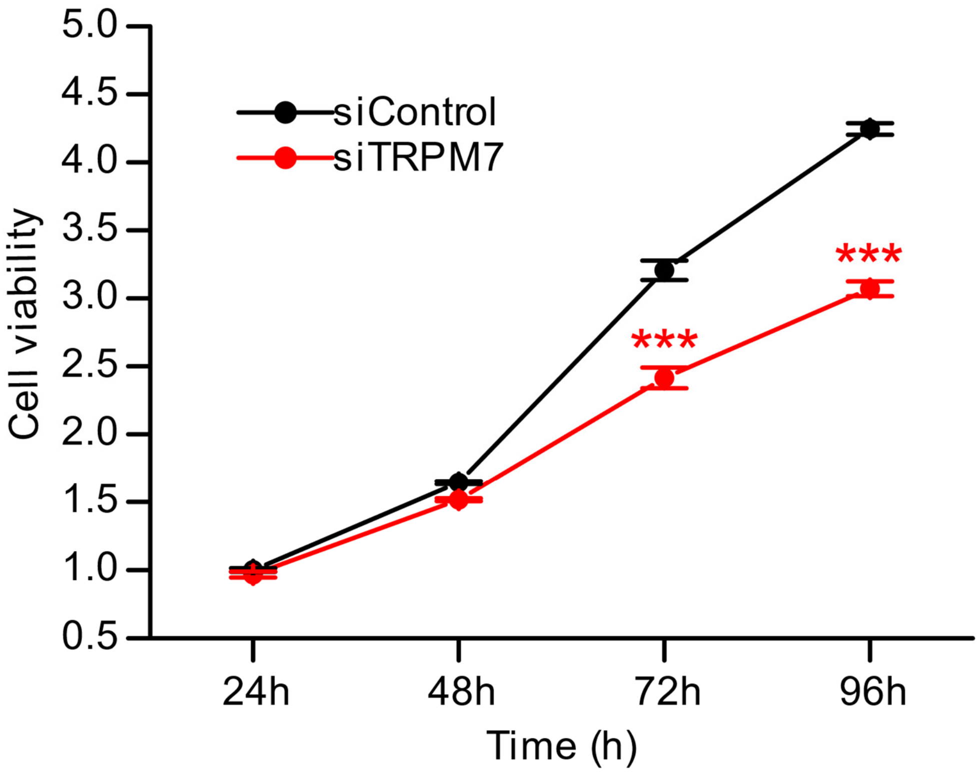

Figure S3: Effect of TRPM7 targeting siRNA on RLT-PSC viability. *** indicates p<0.001 (2-ways ANOVA).
